# EMOTION REGULATION SUCCESS IN A LABORATORY TASK IN YOUNG ADULTS AND COGNITIVELY DIVERSE OLDER ADULTS

**DOI:** 10.1093/geroni/igad104.0110

**Published:** 2023-12-21

**Authors:** Claire Growney, Tammy English

**Affiliations:** Stanford University, Stanford, California, United States; Washington University in St. Louis, St. Louis, Missouri, United States

## Abstract

Age is associated with declines in cognitive resources viewed as important for emotion regulation, and older adults with mild cognitive impairment (MCI) are expected to experience difficulty with emotion regulation. The present study examines how providing support through emotion regulation instructions might contribute to improved emotional well-being using different indices of emotion regulation success. Young adults (age 21–34, n = 66) and older adults with MCI (age 70–84, n = 60) and cognitively normal older adults (age 70–84, n = 90) completed a laboratory task in which they viewed high-arousal negative emotional film clips under instructions to regulate prohdonically (a) using any emotion regulation strategy, and (b) using an instructed strategy based on condition: distraction or reappraisal. We examined three indices of emotion regulation success: self-perceived emotion regulation success, self-reported experience of the target emotion, and behaviorally-coded expression of the target emotion. Overall, there were no group differences in experience or expression, but older adults with MCI perceived that they regulated with less success compared with younger adults and cognitively normal older adults. Younger adults reported experiencing less of the target negative emotion on instructed versus self-selected strategy trials, whereas older adults with MCI expressed less of the target negative emotion on instructed versus self-selected strategy trials. Findings suggest that younger adults and older adults with MCI might benefit more from support with regulating emotions compared with cognitively normal adults, who are theorized to have expertise in this domain.

